# Supplementary material for: Development of a conceptual framework of sexual health for married elderly women: An exploratory mixed method study
Source: PLoS One. 2025 Feb 18;20(2):e0318060. doi: 10.1371/journal.pone.0318060 (PMC11835322; doi:10.1371/journal.pone.0318060)
Supplement: Attachment — Submitted filename: Response to Reviewers.docx [file pone.0318060.s001.docx]

**Dear editor**

Subject: submission of revise paper with ID “PONE-D-24-36737”

I sincerely thank you for the opportunity to edit, revise and present the article again. And many thanks to the reviewers for their valuable comments that were very helpful in improving the writing of the manuscript.

We have carefully reviewed the comments and have revised the manuscript accordingly. Our answers are given point by point below. Changes to the manuscript our shown in highlights (yellow). And the changes in the English version are marked in yellow. We hope revised version is now suitable for publication and looking forward to hearing from you in due course.

Sincerely

Marjan Beigi

**Reviewers’ Comments and Authors Response**

**Dear reviewer 1**

**Thank you for your review of our manuscript. We have answered each of your point below.**

The language can be refined for clarity. Some sentences are lengthy and may benefit from more direct phrasing. Simplify the text to ensure that the key points are easily understood.
Answer: The article was reviewed and revised by a specialized translator. Long sentences were shortened as much as possible.

There are grammatical errors, such as "elimination of agism attitudes" which should be corrected to "elimination of ageist attitudes."

Answer: Grammar errors were corrected by a specialist translator.

The subcategories in each group could be elaborated further to provide more detailed explanations or examples. For instance, what specific strategies or practices might be involved in "eliminating sexual superiority for men" or "enabling enjoyment of sexual pleasure in old age"?
Answer: In the results section, the subcategories were described with more explanations. Strategies were also written. These strategies were clearly emphasized in the discussion section.

Can you elaborate on the criteria used for selecting the studies included in the review?

Answer: Criteria for study selection were explained in the literature review phase (Phase 1).

How do your account for demographic variations (e.g., socioeconomic status, education level) in the final questionnaire?

Answer: The common variables gathered of literature review include age, age gap with spouse, age at marriage, educational level (illiterate, primary, secondary, university), occupation (housewife, employed), and duration of marriage. These were added to the third phase of the study (Phase 3) (before instrument validity).

What specific methods employed to ensure the reliability and validity of the qualitative interviews?
Answer: Validity and reliability of the qualitative phase (interviews) is included as "Rigor and trustworthiness" (after data analysis) (Phase 2).

What ethical guidelines followed to ensure the protection of participants' privacy and confidentiality during qualitative interviews?
Answer: Ethical considerations, especially privacy and confidentiality, were described (Phase 2).

Describe how the 83 studies were identified, including databases searched and search terms used. Specify inclusion and exclusion criteria.
Answer: Inclusion and exclusion criteria, keywords used for searching and databases used were explained in the literature review phase (Phase 1).

Detail the process of extracting data from the selected studies and how this data was categorized.

Answer: The data extraction process was described in Phase 1 of the study. The matrix folder was used to organize the information and the synthesis folder was used to analyze and classify the information. Full details are given in the text of the manuscript (Phase 1).

Provide specifics on the interview process, including participant recruitment, interview structure, and analysis methods. Answer: Interview structure, analysis method, and participant engagement were clarified (phase 2).

Explain the procedures for developing and validating the questionnaire, including pilot testing and statistical analyses to assess reliability and validity.

Answer: Validity and reliability of the questionnaire were adequately explained in the manuscript, including face validity, Initial Test Execution (pilot testing), content validity, criterion validity, construct validity, and reliability measures such as internal consistency and stability. All of these is stated in the manuscript (Phase 3). The titles and subtitles of these parts are highlighted, but their descriptions are without highlights

The discussion section should delve deeper into the implications of the findings. Explore how the study's results compare to existing knowledge, how they advance understanding of elderly sexual health, and how they can be practically applied.
Answer: The discussion was written in depth. Completely revised and formed. The studies were compared.

Consider discussing barriers to implementing the proposed changes in regions with strong cultural resistance, providing insight into how these challenges could be addressed.
Answer: barriers to the implementation of proposed changes in cultures that are resistant were raised in the discussion. Challenges were raised in this regard.

While the study highlights cultural issues specific to Iran and similar contexts, it could benefit from a more balanced discussion that also includes perspectives from different cultural settings. A comparison could enhance the generalizability of the findings.
Answer: Cultural views and comparisons between them were written in the discussion.

Strengthen the argument that the identified demands are deeply rooted in socio-cultural norms by integrating evidence from other studies or cultural analyses. This approach will provide a more comprehensive understanding of how these issues are shaped and perpetuated.
Answer: It was stated in the discussion that the identified demands of elderly women are rooted in cultural norms. Explanations were given.

Emphasize how addressing sexual health demands of elderly women can lead to broader societal benefits beyond individual well-being, such as strengthening family dynamics and improving community health.
Answer: It was clearly noted that addressing elderly sexual health needs can improve family and community health.

The limitations mention that interviews with elderly couples could be beneficial, but this point could be expanded. What specific research questions should be explored in these future studies? How could they potentially impact interventions or policies?

Answer: The potential impact of the suggested study on policies and programs was outlined, and research questions were included.

**Thank you. All the best**

**Dear reviewer 2**

**Thank you for your review of our manuscript. We have answered each of your point below.**

Dear Authors, I liked the hard work that you have put into this work and that you will be doing in the future, however I would like to be more convinced about the importance of your study, especially when you mention policymakers. I think the reader has to really appreciate this much effort that you are putting into an important subject by the rationales that you are giving as important of your study. For me as a reader, I think you have not added strong rationales for your hard work.

Answer: Health policymakers in Iran and in other countries that lack intermediary institutions or supporting non-governmental organizations for health services need such research. In these countries, not only are health-related laws necessarily established by these policymakers, but the process of implementing health programs is also formulated and defined by these same policymakers and legislators.

To clarify the role of politicians, this sentence was added to the text of the manuscript

**Thank you. All the best**
