# Supplementary material for: Development of a conceptual framework of sexual health for married elderly women: An exploratory mixed method study
Source: PLoS One. 2025 Feb 18;20(2):e0318060. doi: 10.1371/journal.pone.0318060 (PMC11835322; doi:10.1371/journal.pone.0318060)
Supplement: Attachment — Submitted filename: Response_to_Reviewers_auresp_2.docx [file pone.0318060.s002.docx]

**Dear editor**

Subject: submission of revise paper with ID “PONE-D-24-36737”

I sincerely thank you for the opportunity to edit, revise and present the article again. And many thanks to the reviewers for their valuable comments that were very helpful in improving the writing of the manuscript. We have carefully reviewed the comments and have revised the manuscript accordingly. Our answers are given point by point below. Changes to the manuscript our shown in highlights (blue). And the changes in the English version are marked in blue. We hope revised version is now suitable for publication and looking forward to hearing from you in due course.

It should be noted that after recent corrections, the article was revised again by an expert translator.

Sincerely

Marjan Beigi

**Reviewers’ Comments and Authors Response**

**Dear reviewer 1**

**Thank you for your review of our manuscript. We have answered each of your point below.**

While the use of STROBE and COREQ checklists is commendable, specifying how these were adapted or applied in detail would strengthen reproducibility and transparency.

Answer: Data extraction was explained in detail by the researchers after evaluating the articles.  Data synthesis and adaptation were explained.

Non-random sampling for quantitative phases may introduce selection bias. Discussing how this limitation will be mitigated (e.g., stratification or weighting during analysis) would add value. Answer: The sampling method is convenient, but to minimize bias, the comprehensive health centers will be considered randomly. This sampling is based on the opinion of a statistician. A full explanation was given in the text.

While Granheim and Lundman’s approach to content analysis is appropriate, elaborating on strategies to ensure consistency among coders (e.g., inter-coder reliability measures) would improve methodological rigor.
Answer: The analysis using the Granheim method was fully explained by two researchers and in coordination with them.

The process for engaging policymakers and ensuring their input into tool development could be clarified. For example, incorporating a Delphi method for expert consensus might add depth to the final recommendations. Answer: The Delphi method will be used exclusively for the fourth phase of the study (prioritizing sexual health needs). A full explanation was given. The first phase (literature review), the second phase (qualitative study), and the third phase (questionnaire development and psychometrics) are carried out exclusively by researchers.

As the study focuses on elderly women within specific cultural contexts (Iranian nationality), discussing how findings might be applicable or adapted to other settings would enhance the study's global relevance. Answer: The generalizability of the study was fully explained.

The use of Moghasemi's questionnaire for concurrent validity is innovative, but discussing potential overlaps and ensuring construct distinction between the tools is essential to avoid redundancy. Answer: Rational reasons were given for comparing the Moghasemi questionnaire with the questionnaire of the present study. This comparison is based on the opinion of a psychometrician.

Including feedback loops after presenting demands to policymakers can ensure that the recommendations are actionable and contextually relevant.

Answer: Interaction and feedback were fully explained.

Plans for the longitudinal assessment of the tool’s impact on sexual health services and outcomes should be considered to validate its practical utility.

Answer: Evaluating the effect of the tool on the sexual health of elderly women is not the focus of this study, but the key points were briefly outlined.

**Thank you. All the best**

**Dear reviewer 2**

**Thank you for your review of our manuscript. All the best**
